# Supplementary material for: Effect of obesity on the acute response to SARS-CoV-2 infection and development of post-acute sequelae of COVID-19 (PASC) in nonhuman primates
Source: bioRxiv. 2025 Feb 22:2025.02.18.638792. Preprint. [Version 2] doi: 10.1101/2025.02.18.638792 (PMC11870618; doi:10.1101/2025.02.18.638792)
Supplement: Supplement 1 [file media-1.pdf]

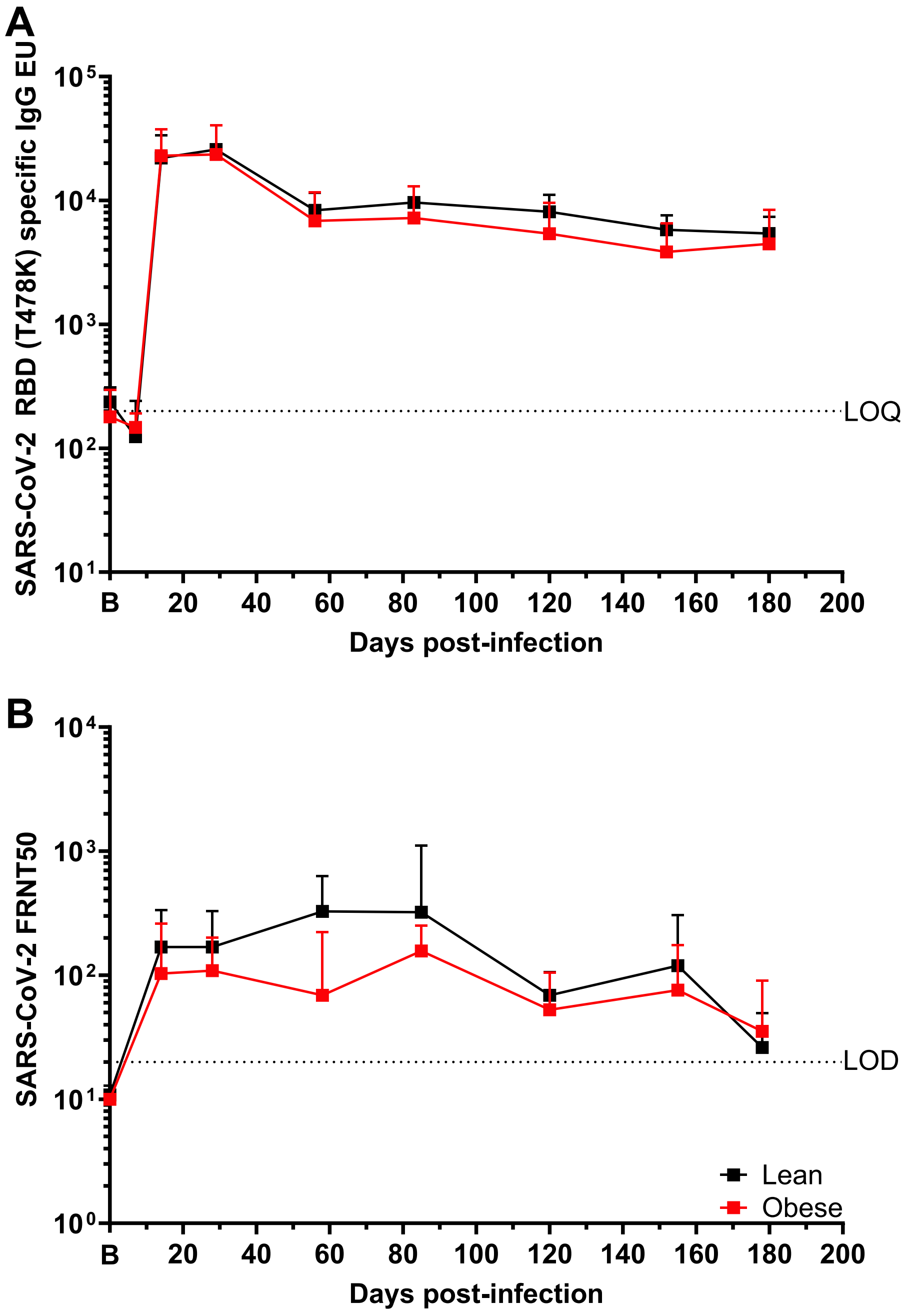

Supplemental figure 1. **SARS-CoV-2-specific serology in lean and obese animals.** A. RBD-binding IgG levels measured by ELISA. B. SARS-CoV-2-specific neutralizing Ab levels. All data are GMT +/-95% CI. LOQ: limit of quantitation. LOD: Limit of detection.
