# Supplementary material for: Effect of obesity on the acute response to SARS-CoV-2 infection and development of post-acute sequelae of COVID-19 (PASC) in nonhuman primates
Source: bioRxiv. 2025 Feb 22:2025.02.18.638792. Preprint. [Version 2] doi: 10.1101/2025.02.18.638792 (PMC11870618; doi:10.1101/2025.02.18.638792)
Supplement: Supplement 6 [file media-6.pdf]

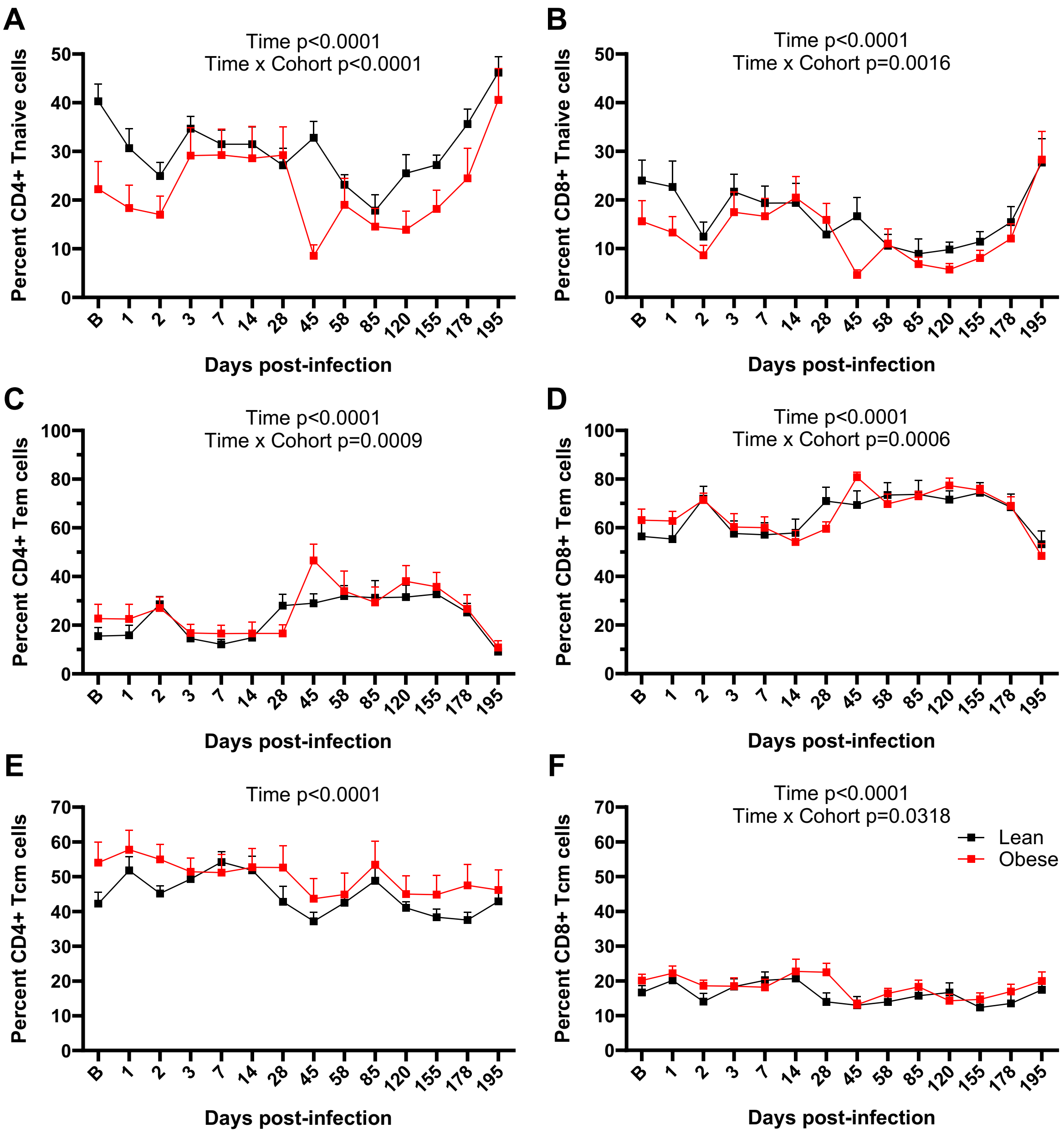

Supplemental figure 6. **T cell memory profiles in lean and obese animals.** PBMCs were analyzed by flow cytometry to distinguish CD4+ naive (A), CD8+ naive (B), CD4+ effector memory (Tem) (C), CD8+ Tem (D), CD4+ central memory (Tcm) (E), and CD8+ Tcm (F) T cells using antibodies and gating strategies shown in Supplemental Table 2 and Supplemental Figure 7. All data are means ± SEM. Significance determined using mixed-effect analysis with Dunnett's post-hoc for multiple comparisons test.
