# Supplementary material for: Effect of obesity on the acute response to SARS-CoV-2 infection and development of post-acute sequelae of COVID-19 (PASC) in nonhuman primates
Source: bioRxiv. 2025 Feb 22:2025.02.18.638792. Preprint. [Version 2] doi: 10.1101/2025.02.18.638792 (PMC11870618; doi:10.1101/2025.02.18.638792)
Supplement: Supplement 7 [file media-7.pdf]

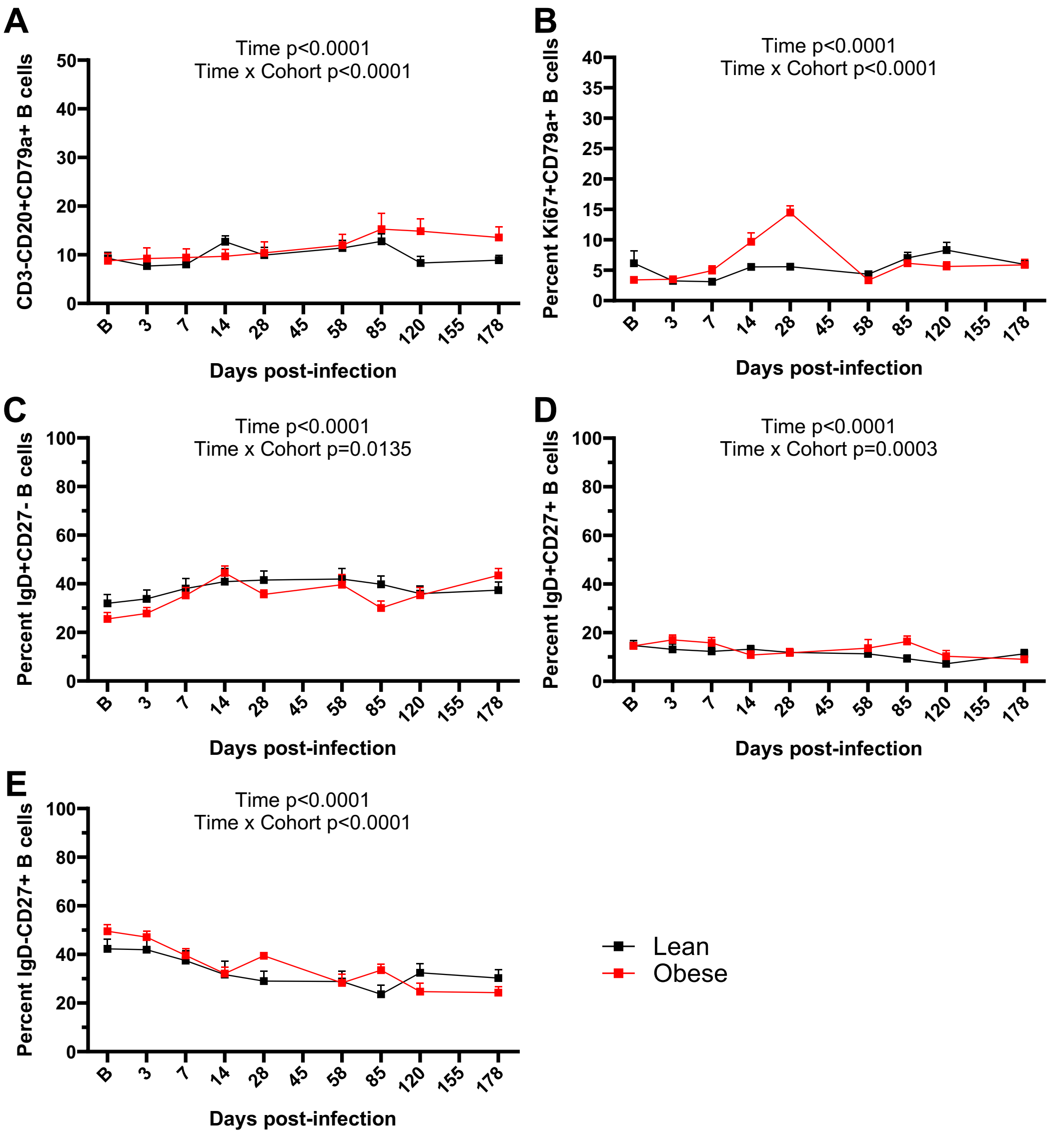

Supplemental figure 7. **B cell profiles in lean and obese animals.** PBMCs were analyzed by flow cytometry to distinguish total CD3-CD20+CD79a+ (A), activated Ki67+CD3-CD20+CD79a+ (B), naïve IgD+CD27- (C), IgM memory IgD+CD27+ (D), and class-switched memory IgD-CD27+ (E) B cells using antibodies and gating strategies shown in Supplemental Table 2 and Supplemental Figure 7. All data are means ± SEM. Significance determined using mixed-effect analysis with Dunnett's post-hoc for multiple comparisons test.
