# Supplementary material for: Effect of obesity on the acute response to SARS-CoV-2 infection and development of post-acute sequelae of COVID-19 (PASC) in nonhuman primates
Source: bioRxiv. 2025 Feb 22:2025.02.18.638792. Preprint. [Version 2] doi: 10.1101/2025.02.18.638792 (PMC11870618; doi:10.1101/2025.02.18.638792)
Supplement: Supplement 8 [file media-8.pdf]

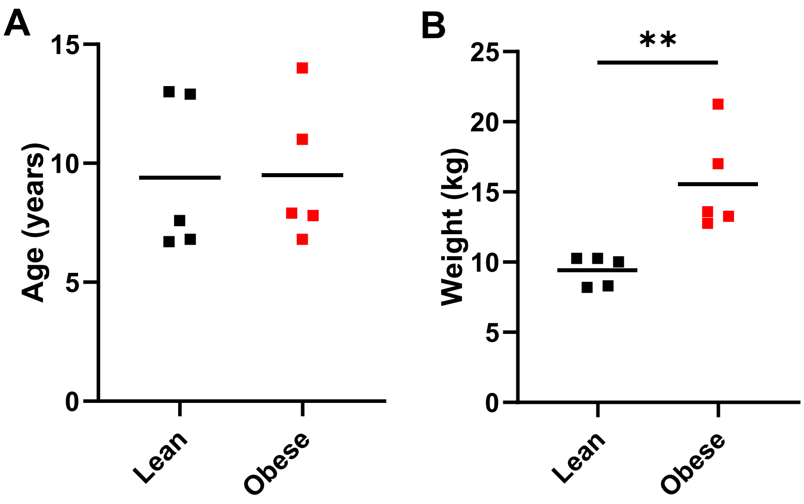

Supplemental figure 8. **Age (A) and weight (B) distribution in animals implanted with telemetry for BT and activity.** Significance was determined by unpaired 2-tailed t test. **, p<0.01.
