# Supplementary material for: Effect of obesity on the acute response to SARS-CoV-2 infection and development of post-acute sequelae of COVID-19 (PASC) in nonhuman primates
Source: bioRxiv. 2025 Feb 22:2025.02.18.638792. Preprint. [Version 2] doi: 10.1101/2025.02.18.638792 (PMC11870618; doi:10.1101/2025.02.18.638792)
Supplement: Supplement 10 [file media-10.pdf]

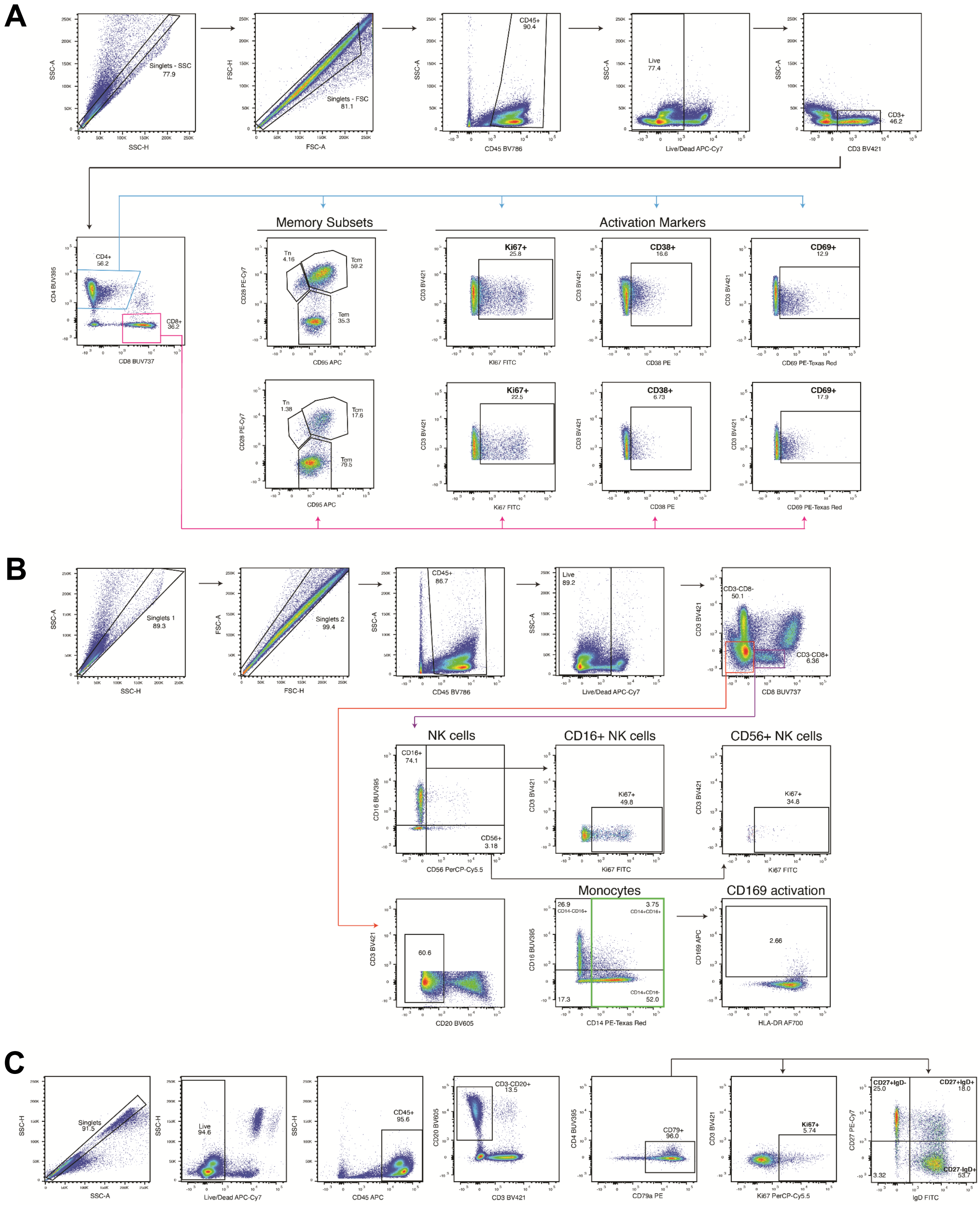

Supplemental figure 10. **Gating strategies for flow cytometry analyses.** PBMCs were characterized for T cells (A), monocytes and NK cells (B), and B cells (C).
