# Supplementary material for: Effect of obesity on the acute response to SARS-CoV-2 infection and development of post-acute sequelae of COVID-19 (PASC) in nonhuman primates
Source: bioRxiv. 2025 Feb 22:2025.02.18.638792. Preprint. [Version 2] doi: 10.1101/2025.02.18.638792 (PMC11870618; doi:10.1101/2025.02.18.638792)
Supplement: Supplement 12 [file media-12.pdf]

Antibody Target	Fluorophore	Clone	Host Species	Source	Catalog #
**CD4**	BUV395	L200	Mouse	BD Biosciences	564107
**CD8**	BUV737	SK1	Mouse	BD Biosciences	612754
**CD45**	BV786	D058-1283	Mouse	BD Biosciences	563861
**CD28**	PE-Cy7	CD28.2	Mouse	BD Biosciences	560684
**CD3**	PB	SP34-2	Mouse	BD Biosciences	558124
**CD95**	APC	DX2	Mouse	BioLegend	305612
**CD38**	PE	OKT10	Mouse	NHPRR	PR-3802
**HLA-DR**	AF700	G46-6	Mouse	BD Biosciences	560743
**CD69**	PE-TR	FN50	Mouse	BioLegend	310942
**Ki67**	FITC	B56	Mouse	BD Biosciences	556026
**CD20**	BV605	2H7	Mouse	BD Biosciences	747736
**CD14**	ECD	RMO52	Mouse	Beckman Coulter	IM2707U
**CD56**	PerCP-Cy5.5	B159	Mouse	BD Biosciences	560842
**CD16**	BUV395	3GB	Mouse	BD Biosciences	563785
**CD169**	APC	7-239	Mouse	Biolegend	346008
**IgD**	FITC	-	Goat	Southern Biotech	2030-09
**CD45**	APC	D058-1283	Mouse	BD Biosciences	561290
**CD27**	PE-Cy7	O323	Mouse	eBioscience	25-0279-42
**CD79a**	PE	HM47	Mouse	eBioscience	12-0792-42
**Ki67**	PerCP-Cy5.5	B56	Mouse	BD Biosciences	561284
**Live/Dead ARD**	APC-Cy7	-	-	Life Technologies	34959

Supplemental table 2. **Antibodies used in flow cytometry.**
